# Assessment of final-year medical students’ entrustable professional activities after education on an interprofessional training ward: A case-control study

**DOI:** 10.1007/s40037-022-00720-0

**Published:** 2022-07-21

**Authors:** Julian Brätz, Lisa Bußenius, Irina Brätz, Hanno Grahn, Sarah Prediger, Sigrid Harendza

**Affiliations:** 1 Heart Center, Cardiology Division, Albertinen Hospital, Hamburg, Germany; 2 III. Department of Internal Medicine, University Medical Center Hamburg-Eppendorf, Hamburg, Germany; 3 Department for Cardiology, University Heart and Vascular Center Hamburg, Hamburg, Germany

**Keywords:** Entrustable professional activities, Interpersonal skills, Interprofessional training ward, Undergraduate medical education

## Abstract

**Introduction:**

Interprofessional training wards (ITWs) are implemented to provide medical students with a holistic and authentic health care experience to improve their clinical competencies. Controlled outcome studies assessing students’ competencies after ITW-training are uncommon. In this case-control study, we assessed final-year medical students who received ITW-training regarding entrustable professional activities (EPAs) and communicative as well as social competencies.

**Methods:**

In March 2021, 32 final-year students, 16 with (ITW group) and 16 without (control group) a previous four-week placement on an ITW participated in a training simulating the first day of residency. The simulated patients assessed students’ communication and interpersonal skills for history taking with the ComCare index after every consultation. Twelve prospective EPAs were assessed by three senior physicians after watching videos of the students’ case presentations.

**Results:**

While baseline characteristics and ComCare index ratings were not significantly different between the two groups, the overall mean entrustment level for the 12 EPAs was significantly higher (*p* < 0.001) in the ITW group compared to the control group (median = 3.15 versus 2.22). The interrater reliability for all EPAs was high and entrustment in students from the ITW group was significantly higher in 10 out of 12 EPAs.

**Discussion:**

ITW training seems to prepare medical students well to practice competencies which are relevant for prospective entrustment decisions and can be deduced by senior physicians from case presentations. Further studies with larger student cohorts are needed to corroborate this finding and observable EPAs could also be defined to assess students’ competencies after ITW training.

**Electronic supplementary material:**

The online version of this article (10.1007/s40037-022-00720-0) contains supplementary material, which is available to authorized users.

## Introduction

With increasing complexity in health care, health professionals need to acquire appropriate competencies to work efficiently and collaboratively in interprofessional teams [1]. These developments require competency-based interprofessional education (IPE) [2], which has been integrated in undergraduate and postgraduate educational frameworks [3, 4]. In a systematic review, positive outcomes of IPE with regard to learners’ satisfaction, their perceptions of other health care professions, and an increase in their collaborative knowledge and skills could be documented [5]. Many countries have established interprofessional training wards (ITWs) for IPE, where learners from more than one profession are responsible for comprehensive patient care [6–8]. Positive outcomes of ITWs with respect to learners’ professional role perception within a health care team and a positive impact on interprofessional collaboration have been described [9]. Additionally, patients’ perceived quality of care on ITWs was high [10] and patient outcomes did not differ between ITWs and usual wards regarding patients’ readmission rates and one-year mortality [11]. ITWs provide excellent opportunities for medical students to be immersed in professional medical activities and to acquire competencies like ‘Responsibility’, ‘Teamwork and collegiality’, ‘Verbal communication with supervisors’, and ‘Knowing and maintaining personal bounds and possibilities’, which are needed for interprofessional care, but also resemble important basic competencies for beginning residents [12, 13] that are relevant for entrustment decisions [14, 15]. However, little is known about medical students’ general performance in competency-based assessments after ITW training.

One possibility to assess medical competencies more holistically is the concept of entrustable professional activities (EPAs) [16]. EPAs integrate different competency domains and provide a construct for training outcomes. Recommendations for precise descriptions of professional activities for EPA-based curricula have been suggested [17] and development processes for EPAs with good content validity and assessment strategies have been described [18, 19]. When used as an assessment framework, EPAs are proposed to best occur in workplace-based situations [20, 21]. Assessment of EPAs was also performed in simulated, experiential situations [22, 23] and can be facilitated by using mobile devices in daily routine [24]. Another approach to assess medical students’ competencies by EPAs was used in competency-based simulations of a first day of residency [14, 25]. Supervisors, who had observed the participating students during a simulated handover of simulated patients before, made prospective entrustment decisions for EPAs, which were unrelated to the content of the simulation [15, 26]. These EPAs were based on basic medical competences relevant for beginning residents [12, 13]. When senior physicians were asked to make prospective entrustment decisions for EPAs based on either watching a 1-minute video sequence of a supervisor greeting a participant at the beginning of the simulation or watching a 1-minute video sequence from the handover of the same participant, no significant differences in EPA assessment were found [27].

In 2020, an ITW for interprofessional education of medical students and nursing trainees was opened at our Medical Center. On this ward, final-year medical students and nursing trainees provide comprehensive patient care for internal medicine patients and are supervised by facilitators of both professions. Basic medical competencies, e.g., ‘*Responsibility*’, ‘*Teamwork and collegiality’, *and ‘*Verbal communication with supervisors*’ [12, 13] were integral part of the ITW’s learning objectives. Communication and interpersonal skills with respect to taking patients’ histories were not part of the ITW curriculum’s specific learning objectives, because communication with patients during history taking is a required skill for all students when they enter the final year of medical school. The aim of this case-control study was to evaluate final-year medical ITW students’ versus control students’ communicative and interpersonal skills by simulated patients as well as prospective EPAs by senior physicians in a telemedicine simulation of a first day of residency. Our hypotheses were: 1) all final-year students communicate equally well with simulated patients, because communication with patients during history taking is not a specific learning objective for final-year students and 2) ITW students will receive higher entrustment scores in prospective EPAs which are based on basic competencies, because basic competencies are a specific part of the ITW curriculum’s learning objectives.

## Methods

### Study design and participants

Thirty-two final-year medical students from the University of Hamburg participated in this case-control study. The study group included 16 students (female: *n* = 7, male: *n* = 9) who had completed the first four months of their final year (mid-November 2020 to mid-March 2021) at the University Medical Center Hamburg-Eppendorf (UKE) in internal medicine. As a mandatory part of their curriculum all 16 students participated in the training on our interprofessional teaching ward (ITW) for four weeks. At the time of their allocation to the UKE, the students were unaware that work on the newly established ITW was part of their internal medicine rotation. The control group included 16 medical students (female: *n* = 11, male: *n* = 5) who completed their final-year internal medicine rotation simultaneously in one of the University of Hamburg’s other teaching hospitals, but received no training on an ITW.

The students were invited to participate in a competency-based telemedicine assessment resembling a first day of residency [28], which took place at UKE in the last week of the students’ internal medicine rotation in March 2021. Students from the ITW group were invited after their ITW rotation by JB, physician and coordinator of the ITW. Students from the control group were invited by e‑mail and included on a first come, first served basis by SP, sociologist and coordinator of the assessment. Participation in the assessment was voluntary and participants provided written consent. All data were anonymized for analysis by the project coordinator SP and consolidated and analyzed by LB. This study was approved by the Ethics Committee of the Chamber of Physicians, Hamburg (*Ethik-Kommission der Ärztekammer Hamburg*, ref. no. PV3649).

### Final-year training in internal medicine

In their final year, medical students must complete a four-month internal medicine rotation, which can be undertaken at the hospital of their choice [29]. Their main tasks on the ward are to draw blood from patients and place intravenous lines, take patients’ histories, and participate in ward rounds and case presentations. Despite the introduction of a National Competence Based Catalogue of Learning Objectives for Undergraduate Medical Education (NKLM) [3], clearly defined learning objectives, independent patient management, and professional supervision are still not provided in general for final-year internal medicine rotations. A pilot project has been started in Germany to introduce a logbook on EPAs in the final year of medical school [30], but this logbook is not available yet for final-year students at the UKE or University of Hamburg’s academic teaching hospitals.

### Training on the ITW

Four medical and four nursing students work together for four weeks on the ITW in interprofessional tandems (one medical student and one nursing trainee). Each interprofessional tandem is responsible for three patients and covers a morning or an afternoon shift each day. The night shift and weekends are covered by physicians and nurses. To prepare the learners for their placement on the ITW they undergo an introductory training on their first day. During this training they get to know the other student members of the team and the supervising professional medical and nursing facilitators. They also learn about relevant aspects of the daily routine on the ITW (e.g., performing a standardized ward round, using the IT systems) and familiarize themselves with technical devices on the ward (defibrillator, monitor system etc.). Besides daily patient care and two supervised interprofessional ward rounds as well as interprofessional handovers between the early and the late shift, the ITW training program focusses on clinical skills and interprofessional tasks including collaborative decisions, treatment and diagnostic planning as well as collaborative admissions and post discharge care. Once a week, a team meeting for bilateral feedback and reflection with all trainees and facilitators takes place. In this context, problematic situations or interactions with patients or colleagues are discussed to eliminate misunderstandings or conflicts and avoid a stereotyping of the professions. Thus, the training program on the ITW includes several of the most important basic medical competencies relevant for beginning residents [12, 13].

### Competency-based telemedicine training

The competency-based telemedicine training [28] was developed due to the COVID-19 pandemic based on a previously validated assessment simulating the first day of residency [25], which was adapted from a validated 360-degree assessment for final-year medical students [14]. The medical students participated in the role of physicians in the simulated clinical routine of a walk-in clinic. Each student was engaged in a consultation hour in a virtual Zoom room (Zoom Video Communications, San José, CA, USA) with four of eight simulated patients, played by professional actors. Afterwards, the students worked with electronic patient charts to document the patients’ history, order diagnostic tests, write medical instructions for the nurses, work up differential diagnoses and develop treatment plans. One of the four patients was assigned to every participant for a digital case presentation round with three other participants and an attending physician. All case presentations and discussions, which lasted 15 min each, took place in a virtual Zoom room and were videotaped.

### Rating instruments and student assessment

#### Assessment of communicative and interpersonal skills with the ComCare index

The simulated patients, who were blinded to the ITW participation status of the students, assessed the participants’ communicative and interpersonal skills after every interview with the validated ComCare index [31]. It consists of three items related to communication (*‘language’, ‘needs’*, and *‘next steps’*) and four items related to interpersonal skills (*‘listening’, ‘interest’, ‘compassion’*, and *‘atmosphere’*), loading on one factor as an overall score. An additional eighth item assesses the satisfaction with the consultation in general. All items are rated on a 5-point Likert scale ranging from 1: full disagreement to 5: full agreement.

#### Prospective assessment of entrustable professional activities (EPAs)

The form for prospective assessment of entrustable professional activities (EPAs) consisted of 12 EPAs [15]. The EPAs were based on different competency domains and did not correspond to activities the participants had to perform in the telemedicine training. Each EPA included a title and a short description of content [32]. The EPA situations included different levels of difficulty. The entrustment for each EPA could be given on a 5-point scale: 1: no permission to act, 2: permission to act with direct supervision, 3: permission to act with direct supervision on demand (supervisor is not in the room, but immediately available), 4: permission to act under distant supervision (i.e., telephone call if necessary), and 5: permission to provide supervision to other trainees. Each participant’s videotaped case presentation was independently watched in randomized order and rated with the EPA assessment form by three trained senior physicians (JB, SH, IB), two of whom (SH and IB) were blinded to the ITW participation status of the students.

### Data analysis

All statistical analyses were performed using IBM SPSS Statistics, version 27 (IBM Corp., Armonk, NY, USA) on a general alpha level of 0.05. Complete data on EPA and ComCare ratings were available for all 32 participants. Mean values for EPA items were calculated by averaging all three raters’ scores. Intraclass correlations (ICC) including 95% confidence intervals (CI) were calculated to examine interrater reliability between all three raters. ComCare values were calculated by averaging all simulated patients’ scores on individual items. To analyse differences in EPA assessment between the ITW and control group we conducted Mann-Whitney U tests on a Bonferroni-corrected alpha level of 0.004, as there were 13 comparisons and data were non-parametric. ComCare performance was also analysed with Mann-Whitney U tests on a Bonferroni-corrected alpha level of 0.006, as there were eight comparisons. Additionally, *r* was calculated to estimate effect sizes.

## Results

A total of 32 students participated in the competency-based assessment in March 2021. The mean age was 27.16 ± 3.09 years and 18 students were female (56.3%). Sixteen students attended the ITW and 16 students completed their internal medicine rotation without training on an ITW. There was no significant difference between the groups in terms of the students’ age (ITW: 26.94 ± 3.32; control: 27.38 ± 2.94, *t*(30) = 0.40, *p* = 0.696) or gender (ITW: 7 females; control: 11 females; χ^2^(1) = 2.03, *p* = 0.154). The assessment of communicative and interpersonal skills by the simulated patients resulted in an overall ComCare mean score of 4.10 ± 0.47 for the entire study group. No significant differences were found between the ITW and the control group for any of the ComCare index items (Table in supplementary material).

The overall entrustment decisions for all participants and the ICC results of the three raters for each EPA are shown in Tab. 1. The highest mean EPA score was reached for EPA 6, *‘Solving a management problem’* (3.21 ± 0.70), the lowest for EPA 12, *‘Acting according to patient’s will’* (1.76 ± 0.62). A good to excellent intraclass correlation between the raters was demonstrated for all 12 EPAs, ranging from 0.77, 95% CI [0.59, 0.88] (EPA 2, *‘Handling a patient’s complaint’*) to 0.96, 95% CI [0.92, 0.98] (EPA 8, *‘Handling a critically ill patient’*).

**Table 1:** Mean EPA ratings and intraclass correlations

EPA	Task	M ± SD	ICC (alpha, 95% CI)
1	Emergency assistance in a case with acute cardiac failure	2.42 ± 0.73	0.91 [0.84, 0.96]
2	Handling a patient’s complaint	2.64 ± 0.54	0.77 [0.59, 0.88]
3	Pre-operative information and consent	3.09 ± 0.66	0.90 [0.83, 0.95]
4	Breaking bad news	2.98 ± 0.80	0.95 [0.91, 0.97]
5	Clinical reasoning under time pressure	2.42 ± 0.59	0.88 [0.78, 0.94]
6	Solving a management problem	3.21 ± 0.70	0.93 [0.88, 0.97]
7	Suspicion of self-induced disease	2.63 ± 0.59	0.83 [0.69, 0.91]
8	Handling a critically ill patient	2.22 ± 0.66	0.96 [0.92, 0.98]
9	Interaction with a consultant	3.17 ± 0.75	0.95 [0.92, 0.98]
10	Presentation of an oncology patient in a tumor board meeting	3.10 ± 0.78	0.92 [0.86, 0.96]
11	Medication error	2.65 ± 0.54	0.84 [0.71, 0.92]
12	Acting according to patient’s will	1.76 ± 0.62	0.87 [0.77, 0.93]

The mean entrustment decision over 12 EPAs, based on video-recorded medical students’ case presentation performance, showed a significantly higher performance of ITW students versus control students (ITW: median = 3.15 vs. control: median = 2.22; *U* = 33.0, *Z* = 3.583, *p* < 0.001, *r* = 0.63) (Tab. 2). Raters entrusted ITW students at a significantly higher level in 10 out of 12 EPAs compared to control students. For EPA 5, *‘Clinical reasoning under time pressure’*, and EPA 8, *‘Handling a critically ill patient’*, no significant differences were seen between the groups. The greatest differences of students’ entrustment levels (∆ means ≈ 1 point on the entrustment scale) were found for EPA 6, *‘Solving a management problem’*, EPA 9, *‘Interaction with a consultant’*, and EPA 10, *‘Presentation of an oncology patient in a tumor board meeting’*, with large effect sizes.

**Table 2:** Group comparison of mean EPA performance

		ITW (*n* = 16)	Control (*n* = 16)	Mann-Whitney U test			
EPA	Task	Median	Median	*U*	*Z*	*p*	*r*
1	Emergency assistance in a case with acute cardiac failure	2.83	2.00	36.5	3.504	< 0.001*	0.62
2	Handling a patient’s complaint	3.00	2.17	39.5	3.411	0.001*	0.60
3	Pre-operative information and consent	3.33	2.67	36.0	3.520	< 0.001*	0.62
4	Breaking bad news	3.67	2.33	41.0	3.381	0.001*	0.60
5	Clinical reasoning under time pressure	2.83	2.00	60.5	2.599	0.009	0.46
6	Solving a management problem	4.00	2.50	32.0	3.782	< 0.001*	0.67
7	Suspicion of self-induced disease	3.00	2.00	51.0	2.969	0.003*	0.52
8	Handling a critically ill patient	2.67	2.00	77.5	2.014	0.044	0.36
9	Interaction with a consultant	4.00	2.50	34.5	3.643	< 0.001*	0.64
10	Presentation of an oncology patient in a tumor board meeting	4.00	2.33	34.0	3.746	< 0.001*	0.66
11	Medication error	3.00	2.17	43.0	3.272	0.001	0.58
12	Acting according to patient’s will	2.00	1.17	40.0	3.389	0.001	0.60
	Mean	3.15	2.22	33.0	3.583	< 0.001*	0.63

*p < 0.004

## Discussion

While we found no differences between the two groups of final-year students with regard to their communicative and interpersonal skills during history taking, students from the ITW group received significantly higher entrustment scores for 10 of the 12 EPAs than those of the control group. The largest differences and effects were found for EPA 9, *‘Interaction with a consultant’*, and EPA 10, *‘Presentation of an oncology patient in a tumor board meeting’*, which have been shown to correlate well with the competency facet *‘Verbal communication with colleagues and supervisors’* [15]. This competency was ranked highly when physician educators were asked to assess the relevance of different competencies for beginning residents [12]. When final-year medical students in Germany were asked to self-assess their competency with respect to ten competencies relevant for beginning residents, they rated their competence level for this facet on average the second lowest [33]. We assume that the students in our study, who were placed on the ITW, gained a lot of experience in verbal communication with colleagues and supervisors because presentation of their patients to the team was an explicit part of their daily work routine on the ITW and could have led to higher entrustment levels in the above-mentioned EPAs.

The competency *‘Responsibility’* was represented in ten, the competency *‘Structure, work planning and priorities’* in eleven of the twelve EPAs we used in our prospective assessment [15]. Responsibility has been found to be a key ingredient of reliability that clinical supervisors find important when making entrustment decisions [34]. Additionally, responsibility was found to be the competency facet with the highest priority for residency training as defined by clinical supervisors from different medical schools [13] and by medical students themselves [35]. Providing learners with opportunities to practice taking responsibility was suggested for the implementation of EPAs for assessment [36]. Such opportunities were presented on a daily basis to the students from our ITW group while caring for their patients on the ITW. The curriculum of the students from the control group did not provide such possibilities and therefore might have led to lower EPA scores. Better EPA scores in the ITW group could be related to the competency *‘Structure, work planning and priorities’*. Its performance level is rated as one of the lowest in final-year students’ self-assessment [33], while it is regarded to be highly important for residency by physicians [13] and medical students [35]. Students on our ITW are constantly in the position to prioritize and structure their work according to the demands of the patients’ situation. This could have led to a good structure in the patient handovers, which represented the basis for the assessors’ prospective entrustment decisions.

The communicative and interpersonal skills, measured by the simulated patients in our study with the ComCare index after history taking [31], did not significantly differ between the ITW and the control group. Communication competency with patients is gained longitudinally at our medical school [37] according to recently published guidelines [38]. It is not a particular learning focus on our ITW, which could be a reason for not finding any differences. Furthermore, the results for the prospective EPAs 5, *‘Clinical reasoning under time pressure’*, and 8, *‘Handling a critically ill patient’*, showed very low overall entrustment scores and also did not significantly differ between ITW and control group. Both EPAs require entrustment in time-critical performance that could compromise patient safety if not delivered. This is neither taught in general in our curriculum nor was it taught on the ITW, where facilitators took over in critical situations, which also underscores our hypothesis that the differences in entrustment scores we discovered could be related to specific competencies taught on the ITW.

However, this finding could also be due to the raters using the prospective assessment scale incorrectly [39] with the observed handover performance in mind and the knowledge that timely reactions to critically ill patients are not a learning objective in the final year of medical students’ training. This would be a limitation to this study. It has been shown that prospective rating scales bear challenges to the raters because they have to deduce entrustment, for instance, for situations they did not observe [40]. Even though our raters received a frame of reference training for prospective assessment [14], this bias could have occurred. Another limitation with respect to the EPA rating is that one of the three raters was not fully blinded to the ITW participation status of the participating students. However, good interrater reliability between the three independent raters was reached. This study was performed at only one medical school and the study sample was small, which limits the generalizability of our results. Furthermore, the control group participants were chosen on a first come, first served basis. This could have led to a self-selection bias of very skilled or motivated students. Despite this limitation, differences in entrustment decisions were large between the two groups. A strength of our study is that we used a control group design to assess outcomes of final-year medical students after working on an ITW, whereas other studies used ITW groups only [41, 42]. Another strength is that we used rater-based assessment for competency measurements performed by senior physicians and simulated patients rather than self-reports on competencies by medical students who participated in ITW training [43–45].

In conclusion, final-year medical students in our study who were trained on an ITW showed similar communicative and interpersonal skills in simulated patient interviews compared to a control group without ITW training. The ITW students received significantly higher entrustment scores in assessment of prospective EPAs based on raters’ observations of a simulated patient handover. To test our hypothesis that this difference in entrustment is related to basic medical competencies that are taught on the ITW as part of the interprofessional training, assessment of the respective competencies or core EPAs which include these competencies could be performed by direct workplace or simulation-based observation.

## Supplementary Information



