# Supplementary material for: Assessment of final-year medical students’ entrustable professional activities after education on an interprofessional training ward: A case-control study
Source: Perspect Med Educ. 2022 Jul 21;11(5):266–72. doi: 10.1007/s40037-022-00720-0 (PMC9302559; doi:10.1007/s40037-022-00720-0)
Supplement: Supplementary file 1 [file 40037_2022_720_MOESM1_ESM.docx]

**Table 1** Group comparison of mean ComCare performance

| **ComCare** |  | **ITW**  **(*n* = 16)** |  | **Control**  **(*n* = 16)** |  | **Mann-Whitney U test** | | | |
| --- | --- | --- | --- | --- | --- | --- | --- | --- | --- |
|  |  | Median |  | Median |  | *U* | *Z* | *p* | *r* |
| Language |  | 4.75 |  | 5.00 |  | 112.5 | -0.638 | 0.523 | 0.11 |
| Listening |  | 4.50 |  | 4.38 |  | 121.0 | 0.268 | 0.789 | 0.05 |
| Interest |  | 3.25 |  | 3.63 |  | 124.0 | -0.152 | 0.879 | 0.03 |
| Needs |  | 4.13 |  | 4.25 |  | 107.5 | 0.780 | 0.435 | 0.14 |
| Compassion |  | 3.88 |  | 4.00 |  | 109.5 | -0.702 | 0.483 | 0.12 |
| Next steps |  | 4.50 |  | 4.50 |  | 119.0 | 0.347 | 0.728 | 0.06 |
| Atmosphere |  | 4.00 |  | 4.00 |  | 119.5 | 0.323 | 0.747 | 0.06 |
| **Total** |  | 4.15 |  | 4.21 |  | 126.0 | -0.075 | 0.940 | 0.01 |
| Satisfaction |  | 4.13 |  | 4.25 |  | 125.5 | 0.096 | 0.924 | 0.02 |
